# Peripheral MC1R activation modulates immune responses and is neuroprotective in a mouse model of Parkinson’s disease

**DOI:** 10.21203/rs.3.rs-3042571/v1

**Published:** 2023-06-12

**Authors:** Pranay Srivastava, Shuhei Nishiyama, Sonia H Lin, Akriti Srivastava, Chienwen Su, Weiyi Peng, Michael Levy, Michael Schwarzschild, Yuehang Xu, Xiqun Chen

**Affiliations:** MassGeneral Institute for Neurodegenerative Disease, Department of Neurology, Massachusetts General Hospital, Harvard Medical School; MassGeneral Institute for Neurodegenerative Disease, Department of Neurology, Massachusetts General Hospital, Harvard Medical School; MassGeneral Institute for Neurodegenerative Disease, Department of Neurology, Massachusetts General Hospital, Harvard Medical School; MassGeneral Institute for Neurodegenerative Disease, Department of Neurology, Massachusetts General Hospital, Harvard Medical School; MassGeneral Institute for Neurodegenerative Disease, Department of Neurology, Massachusetts General Hospital, Harvard Medical School; Department of Biology and Biochemistry, University of Houston; MassGeneral Institute for Neurodegenerative Disease, Department of Neurology, Massachusetts General Hospital, Harvard Medical School; MassGeneral Institute for Neurodegenerative Disease, Department of Neurology, Massachusetts General Hospital, Harvard Medical School; MassGeneral Institute for Neurodegenerative Disease, Department of Neurology, Massachusetts General Hospital, Harvard Medical School; MassGeneral Institute for Neurodegenerative Disease, Department of Neurology, Massachusetts General Hospital, Harvard Medical School

**Keywords:** BBB, LPS, Melanocortin 1 receptor, MPTP, Parkinson’s disease, Regulatory T cells (Tregs)

## Abstract

**Background:**

Melanocortin 1 receptor (*MC1R*) is a key pigmentation gene, and loss-of-function of *MC1R* variants that produce red hair may be associated with Parkinson’s disease (PD). We previously reported compromised dopaminergic neuron survival in *Mc1r* mutant mice and dopaminergic neuroprotective effects of local injection of a MC1R agonist to the brain or a systemically administered MC1R agonist with appreciable CNS permeability. Beyond melanocytes and dopaminergic neurons, MC1R is expressed in other peripheral tissues and cell types, including immune cells. The present study investigates the impact of NDP-MSH, a synthetic melanocortin receptor (MCR) agonist that does not cross BBB, on the immune system and the nigrostriatal dopaminergic system in mouse model of PD.

**Methods:**

C57BL/6 mice were treated systemically with MPTP^.^HCl (20 mg/kg) and LPS (1 mg/kg) from day 1 to day 4 and NDP-MSH (400 μg/kg) or vehicle from day 1 to day 12 following which the mice were sacrificed. Peripheral and CNS immune cells were phenotyped and inflammatory markers were measured. The nigrostriatal dopaminergic system was assessed behaviorally, chemically, immunologically, and pathologically. To understand the role of regulatory T cells (Tregs) in this model, CD25 monoclonal antibody was used to deplete CD25+ Tregs.

**Results:**

Systemic NDP-MSH administration significantly attenuated striatal dopamine depletion and nigral dopaminergic neuron loss induced by MPTP+LPS. It improved the behavioral outcomes in the pole test. *Mc1r* mutant mice injected with NDP-MSH in the MPTP and LPS paradigm showed no changes in striatal dopamine levels suggesting that the NDP-MSH acts through the MC1R pathway. Although no NDP-MSH was detected in the brain, peripheral, NDP-MSH attenuated neuroinflammation as observed by diminished microglial activation in the nigral region, along with reduced TNF-α and IL1β levels in the ventral midbrain. Depletion of Tregs limited the neuroprotective effects of NDP-MSH.

**Conclusions:**

Our study demonstrates that peripherally acting NDP-MSH confers protection on dopaminergic nigrostriatal neurons and reduces hyperactivated microglia. NDP-MSH modulates peripheral immune responses, and Tregs may be involved in the neuroprotective effect of NDP-MSH.

## Background

Melanocortin receptors (MCRs) are a family of five G-protein coupled receptors. Among them, melanocortin 1 receptor (MC1R) is expressed in melanocytes and regulates pigmentation of the skin and hair. Upon binding to its ligand alpha-melanocyte stimulating hormone (α-MSH), MC1R activates the cAMP pathway and facilitates the synthesis of brown/black pigment eumelanin, increasing the ratio of red/blonde pheomelanin to eumelanin (1,2). Red hair and fair skin in people are usually due to loss-of-function *MC1R* variants and are associated with accelerated skin aging, as well as increased melanoma risk (2–4). Red hair and *MC1R* loss-of-function variants have also been reported to be associated with increased risk for Parkinson’s disease (PD), a common neurodegenerative disease that has been consistently linked to melanoma (5).

Pathologically, PD is characterized by loss of dopaminergic neurons in the substantia nigra (SN) of the brain and abnormal accumulation and aggregation of alpha-synuclein (αSyn) in the nervous system. Although the etiology of PD is unclear, oxidative stress, mitochondrial dysfunction, neuronal network alteration, and neuroinflammation have been reported to be important contributors (6). Additionally, there is mounting evidence that chronic systemic inflammation (7)with the accompanying dysregulation of circulating inflammatory molecules and the innate immune response, play prominent roles in PD(8). It is increasing appreciated that peripheral, as well as brain inflammation, contribute to the onset and progression of the neurodegenerative processes in PD (9). Previous studies from our group reported expression of MC1R in dopaminergic neurons (10). *Mc1r* mutant mice showed a compromised dopaminergic system with greater susceptibility to PD-associated mitochondrial toxin MPTP (1-methyl-4-phenyl-1,2,3,6-tetrahydropyridine) and αSyn overexpression, whereas locally administered MCR agonist NDP-MSH ([Nle^4^, DPhe^7^]-α-MSH) attenuated αSyn toxicity in brain of naïve mice (10–12). NDP-MSH is a synthetic analog to α-MSH and is chemically more stable than α-MSH. Similar to α-MSH, NDP-MSH can activate all MCRs, though its affinity is highest for MC1R. NDP-MSH is not detectable in the brain after systemic i.p. injection in C57BL/6 mice (12). Both α-MSH and NDP-MSH (13) have additionally been shown to protect in models of other CNS disorders after systemic administration, including ischemic stroke, spinal cord injury, traumatic brain injury, Alzheimer’s disease (AD), and other neuroinflammation-associated diseases like intracerebral hemorrhage (14–18). The direct site of the NDP-MSH actions in these models was either known to include CNS due to obvious blood brain barrier (BBB) disruption (17) or not specifically characterized (15).

In addition to regulating pigmentation and other cellular functions in melanocytes, MC1R is present in immune cells like CD4^+^ T cells, and monocytes (19–21) and involved in modulating immune responses and inflammation (21,22). Abnormal immune and inflammatory responses have emerged as prominent factors potentially underlying onset and progression of PD. For example, persistent microglia activation has been well characterized. Besides microglia, another population of immune cells implicated in disease pathogenesis consists of monocytes, which also expresses MC1R (23). An analysis of myeloid compartment in PD patients revealed migration of peripheral monocytes to the CNS that was in sync with rodent studies (24,25). Dopaminergic neurons in SN is highly sensitive to pro-inflammatory cytokines like TNF-α and IFNγ in models of PD (26–28). Growing evidence also suggests a role of perturbed peripheral immune components and chronic inflammatory cascades in the pathophysiology of PD (8). Populations of peripheral lymphoid cells including CD4^+^ helper T cell, and CD8^+^ cytotoxic T cells were altered in PD patients (29,30).

Regulatory T cells (Tregs), forming the immunosuppressive T-cell subset, are involved in maintaining immune homeostasis. Dysregulated Tregs can cause increased levels of proinflammatory mediators leading to exacerbated immune responses in PD (31–36) and restoration of Treg function has been proposed to have therapeutic implications (37). Reynolds et al. demonstrated a perturbed nigrostriatal dopaminergic system associated with dysfunctional Tregs in an αSyn immunization PD model (19). Expansion of Treg populations is the mechanism behind the protection by α-MSH and NDP-MSH in experimental autoimmune encephalomyelitis (EAE) acting through MC1R expressed on T cells (17).

The present study investigates the effects of the systemic melanocortin activator NDP-MSH in the MPTP mouse model of PD, with systemic inflammation induced by lipopolysaccharide (LPS). LPS has been shown to have poor BBB penetrance, especially at lower doses (38,39) and systemic LPS likely causes neuroinflammation indirectly (40). LPS also activates CD4+ cells, monocytes, and neutrophils and significantly increases the circulating cytokines TNF-α , IL-1β, etc (41,42). PD patients exhibit an impaired immune response with the contribution of monocytes, CD4+ cells, regulatory T cells, and increased circulatory cytokines (TNF, IFNγ, IL-1β, IL-6, IL-2) (9). Presence of systemic inflammation in PD patients along with MCRs on immune cells prompted us to utilize systemic inflammatory approach with LPS. Immune responses and the integrity of the nigrostriatal dopaminergic system were assessed following NDP-MSH treatment. The role of Tregs was characterized by antibody-mediated depletion of CD25+ Tregs.

## Material and methods

### Animals

C57BL/6J mice (3–4 months old) were purchased from Jackson Laboratory (Bar Harbor, ME). Male mice were used due to ~80–100% MPTP mortality in female mice based on our observations. Mice were kept in a temperature-controlled room, with a 12-h light/dark cycle, and had free access to food and water. To assess the MC1R-dependence of effects, Mc1r^e/e^ mice (3–4 months old) were used. Mc1r^e/e^ mice carry an inactivating mutation of *Mc1r* in a C57BL/6J background. All procedures were approved by the Institutional Animal Ethical Committee of Massachusetts General Hospital (animal protocol # 2018N000039).

### Chemicals and treatment paradigms

García-Domínguez et al. reported an enhanced microglia response and nigral dopaminergic cell death in an acute MPTP model following peripheral inflammation resulting from single i.p. injection of LPS at 2 mg/kg (43). We employed a subacute paradigm to inject lower doses of MPTP^.^HCl (20 mg/kg) and LPS (1 mg/kg) over 4 days to induce systemic inflammation. Mice were randomly divided into MPTP+LPS+NDP-MSH, MPTP+LPS, and control groups to receive i.p. once daily MPTP^.^HCl (Millipore Sigma, Cat# M0896; 20 mg/kg) or saline and LPS (Millipore Sigma, Cat# L4391; 1 mg/kg) or PBS from day 1 to day 4. NDP-MSH (Genscript, Cat# RP10658; 400 μg/kg) or PBS was injected from day 1 to day 12. Mice were tested for behavioral activities and were sacrificed thereafter on day 12.

To study the role of Tregs, animals were treated with anti-mouse CD25 monoclonal antibody (clone PC61, Biolegend, Cat# 102059; 400 μg/mouse) or isotype control (Biolegend, Cat# 401916; 100μg/mice) for 3 alternate days, 1 week before the start of experiment. Mice were subsequently treated with MPTP, LPS and NDP-MSH as described above. Another dose of anti-mouse CD25 monoclonal antibody or isotype control was administered 2 days before the sacrifice.

### BBB permeability and NDP-MSH pharmacokinetics (PK) study

The integrity of BBB was measured through FITC-albumin (Millipore Sigma, Cat# A9771) leakage from vasculature into brain parenchyma as described previously (44). Mice were treated with MPTP+LPS and sacrificed after 6 h and 24 h after the last dose. Briefly, mice were anaesthetized by isoflurane and perfused intracardially with heparin (100 units/kg) followed by 5 ml FITC albumin at a concentration of 5 mg/ml in PBS with a flow rate of 1.5 ml/min. Subsequently, the brain was isolated and incubated in 4% paraformaldehyde overnight. The solution was changed to 30% sucrose in PBS. Coronal sections of striatum were mounted and analyzed under fluorescence microscope (Olympus BX51 microscope).

To assess NDP-MSH concentrations in plasma and brain, male mice were treated with MPTP+LPS as described above and two concentrations of NDP-MSH (400 μg/kg and 1 mg/kg) and sacrificed after 5-, 30- and 90-min. Blood samples were collected through cardiac puncture in 40 mM EDTA, and plasma was collected by centrifugation and stored at −80°C till further analysis. Whole brain was dissected and homogenized in PBS. Proteins in brain homogenate and plasma samples were crashed with 3 volumes of methanol containing internal standard (propranolol) and centrifuged. Supernatants were analyzed by liquid chromatography/mass spectrometry (LC/MS). NDP-MSH in plasma and brain samples was detected by LC/MS through a service contract with Cyprotex, LLC, MA, USA.

### Open field test

Locomotor activity was determined at the baseline and post treatment by open field test. Briefly, the mice were placed in the plexiglass chamber (11 × 11 in with clear 8-in high walls) and were allowed to explore for a period of 10 min. The total distance travelled was measured with software Ethovision XT 9.0, Noldus Information Technology, The Netherlands.

### Pole test

Pole test was performed at the baseline and post treatment to test motor coordination and motor abnormalities that result from depletion of striatal dopamine. Mice were trained on the pole (1 cm diameter, 50 cm height) one day before the start of the experiment for 120s. Time taken by the mice to turn (T turn) and time taken to climb down (T descent) the pole were recorded (45,46).

### Immunohistochemistry and stereological counting of SN dopaminergic neurons

Immunohistochemistry was performed on the coronal sections of SN as described previously (12). In brief, the 30μm sections were incubated in blocking solution (10%, normal goat serum) for 1h followed by incubating them with either of the following primary antibodies (Enzo Life Sciences, Cat# BML-SA497–0100, Tyrosine Hydroxylase (TH) (1:1000); Abcam, Cat# ab178847, Ionized calcium binding adaptor molecule 1 (iba1) (1:500); Biolegend, Cat# 840001, Glial Fibrillary Acidic Protein (GFAP) (1:500) overnight at 4°C. For peroxidase staining, sections were incubated with biotinylated secondary antibodies (Millipore, Cat# OS03B, anti-rabbit 1:2000); Sigma-Aldrich, Cat# B7264, anti-mouse 1:2000) followed by incubating in avidin biotin complex (Vector laboratories, Cat# PK6100) and the staining was developed by incubation in 3,3′-diaminobenzidine (DAB) (Millipore Sigma, Cat# D4418). TH, iba1, and GFAP are markers for dopaminergic neurons, microglia, and astrocytes, respectively.

Stereological counting of SN TH+ cells was performed to determine the total number of dopaminergic neurons in the SN as previously described (12). In brief, a complete set of coronal midbrain sections stained with TH and counterstained with Nissl was counted stereologically with Olympus BX51 microscope and Olympus CAST stereology software.

The method published by Sanchez-Guajardo et al. (2010) was referred to for analysis and classification of morphology of iba1+ microglia cells in SNpc (47). These cells can be classified according to their morphology into resting type (type A with a thin and visible cytoplasm with long and thin processes), activated type (type B with thick and short processes extending from a dense and enlarged cell body), and phagocytic type (type C with a shape resembling pseudo-amoeba, a big and dark cell body with processes). The stereological method was followed to count the cells at 40× magnification (Olympus BX51 microscope and Olympus CAST stereology software) as previously described by Dimant et al., 2013 and West et al., 1991(48,49). Two midbrain sections with the central and anterior SN were analyzed per mouse.

Integrated optical density of GFAP immunoreactivity was determined by Image J as a measurement of astrogliosis. The images were captured using ×40 objective. Two midbrain sections with the central and anterior SN were analyzed for each mouse. The general protocol used for TH cell staining was deposited in protocols.io (DOI: dx.doi.org/10.17504/protocols.io.j8nlk4yw1g5r/v1)

### High-performance liquid chromatography

High-performance liquid chromatography with electrochemical detection (HPLC-ECD) was used to measure striatal dopamine levels as previously described (50,51). Briefly, the striatum was dissected from the brain, homogenized in buffer containing perchloric acid and centrifuged at 16000g for 20 min followed by analysis of the supernatant through HPLC-ECD. The general protocol used for measurement of dopamine was deposited in protocols.io (DOI: dx.doi.org/10.17504/protocols.io.dm6gpbjdplzp/v1).

Additionally striatal 1-methyl-4-phenylpyridinium (MPP+) was measured 90 min and 6 h after the last dose of MPTP. Striatum was dissected and analyzed by HPLC ultraviolet-ultraviolet (UV) detection as previously described (52).

### ELISA

Levels of IL-1β and TNF-α in plasma and brain tissue were determined by ELISA as described previously (53). In brief, blood samples from all the treatment groups were collected through cardiac puncture in 40 mM EDTA and plasma was collected by centrifugation. The plasma samples were immediately transferred to dry ice and then stored at −80°C till further analysis. A small fraction of ventral midbrain tissue homogenate prepared in 1x RIPA buffer (Cell Signaling, Cat#9806) was used for analysis of IL-1β and TNF-α using mouse ELISA kits (Biolegend Cat# 430904; 432604).

### Flow cytometry

To assess immune cell profile, single-cell suspension of spleen tissue was prepared according to the protocol described previously (54). Spleen was removed in a 35 mm petri plate with 5 ml RPMI 1640 and digested mechanically and passed through 70 μm filter screen. The cell suspension was centrifuged and the pellet was incubated in RBC lysis buffer. The resulting cell suspension was washed in 1xPBS and blocked with Fc Block (Biolegend, Cat# 101302, 1 μl/50 μl). The cells were incubated with MC1R (Invitrogen, Cat# PIPA521911, 1.39 μg) antibody followed by fluorophore conjugated primary antibodies for extracellular markers (Biolegend Cat# 101235, CD11b-BV421(0.25 μg); Cat# 127641, Ly6G-BV-650 (0.25 μg); Cat# 128041, Ly6C-BV785 (0.125 μg); Cat# 100516, CD4-APC (0.25 μg); Cat# 100751, CD8a-BV510 (0.5 μg); Cat# 152405, CD19-PerCP-Cy5.5 (0.25 μg); Cat# 102036, CD25-BV605 (0.3 μg)) and AF488 (Invitrogen, Cat# A11034, 1:200). Zombie dye (Biolegend, Cat# 423101, 1μl/sample) was used to differentiate between live and dead cells. Helper T cells and cytotoxic T cells were identified by CD4+ and CD8+, respectively. CD4+CD25+ cells were used to mark Tregs. CD19+ cells were used as marker for B cells. Monocytes were identified as CD11b+Ly6G-Ly6C^high^ cells and neutrophils were marked by CD11b+Ly6C-Ly6G+. For monocytes and neutrophils, CD11b-positive cells were first extracted from the live cell subset by expansion with SSC-A, followed by Ly6C. After expansion with Ly6C and Ly6G, we excluded Ly6G-positive cells. Monocytes were identified as CD11b+Ly6G-Ly6Chigh cells, and neutrophils were marked by CD11b+Ly6C-Ly6G+. SORP 5 Laser BD Fortessa X-20 (BD Bioscience) and FlowJo v10.7.1 (Becton Dickson & Company) software was used for data acquisition. Single cell control and manual compensation was used for gating strategy. Abundance of the cell population was calculated and presented as % of the population relative to live cells. FlowJo v10.7.1 (Becton Dickson & Company) software was used for data analysis.

### Statistical analysis

Data from each experiment was represented as mean ± SEM and statistical significance was determined by One-way ANOVA with Tukey post hoc test. Two-way ANOVA with Tukey post hoc test was used to analyze neurobehavioral endpoints in open field test and pole test to compare baseline and post treatment effects. GraphPad Prism 8.3.0 (GraphPad Software, San Diego, CA, USA) was used to analyze the data.

## Results

### Systemically administered NDP-MSH ameliorated behavior impairment and protected dopaminergic neurotoxicity in MPTP and LPS mouse model of PD

**Figure 1:**
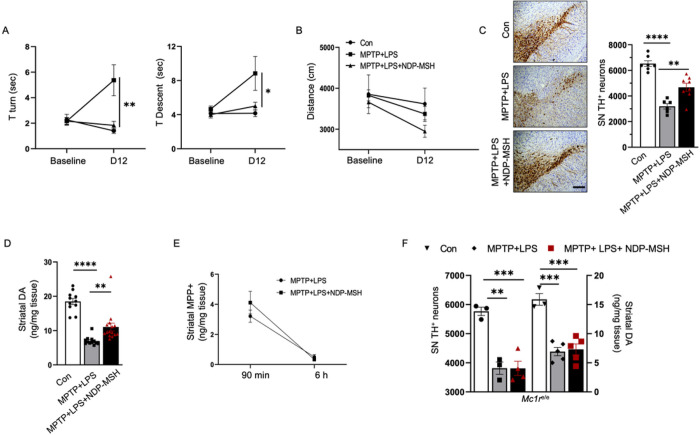
Systemic NDP-MSH treatment protects against MPTP+LPS-induced dopaminergic neurotoxicity. C57BL/6J mice were treated i.p. with MPTP^.^HCl (20 mg/kg) and LPS (1 mg/kg) or vehicle (Con) from day (D) 1 to D4 and NDP-MSH (400 μg/kg) or vehicle from D1 to D12 and sacrificed at D12. (A) Pole test for the time taken to turn downward (T turn) on the top of a pole and time taken to climb down (T descent) and (B) open field test to measure the total distance traveled. Two-way ANOVA by Tukey’s post hoc test. *p<0.05, **p<0.01 for MPTP+LPS vs MPTP+LPS+NDP-MSH; n=5–7/group. (C) Representative micrograph and stereological quantification of TH+ cells in SN; n=6–8/group. Scale bar, 100 μm. (D) Striatal dopamine content; n=11–15/group. (E) Striatal MPP+ levels assessed at 90 min and 6 h after treatment with MPTP+LPS with or without NDP-MSH. Two-way ANOVA followed by Tukey’s post hoc test, n=3/group. *Mc1r*^*e/e*^ mice carrying non-functioning Mc1r were treated with the same paradigm. (F) Stereological quantification of TH+ cells in SN (left y-axis) and striatal dopamine content (right y-axis); n=3–5/group. One-way ANOVA followed by Tukey’s post hoc test. **p<0.01; ***p<0.001

We used MPTP and LPS to introduce a PD-like phenotype in the context of systemic inflammation to assess effects of NDP-MSH on behavior and the nigrostriatal dopaminergic pathway. NDP-MSH treatment in MPTP+LPS exposed mice exhibited improved behavior as they took less time to turn around and climb down in the pole test as compared with MPTP+LPS group treated with vehicle (Figure 1A). No significant difference in distance traveled in open field was observed among the three groups (Figure 1B).

Stereological counting of TH+ neurons demonstrated more SN TH+ cells were preserved in MPTP+LPS mice treated with NDP-MSH compared with vehicle treated MPTP+LPS mice (Figure 1C). Similarly, higher striatal dopamine levels were observed in MPTP+LPS mice treated with NDP-MSH compared with vehicle treated MPTP+LPS mice (Figure 1D).

Following systemic administration, MPTP gets transported to the brain, metabolized into its active form MPP+ that is toxic and causes death of dopaminergic neurons (55). There was no difference in MPP+ levels in the striatum between NDP-MSH vs vehicle treated MPTP+LPS mice at 90 min and 6 h after the last dose, suggesting that NDP-MSH treatment is not associated with altered MPTP metabolism (Figure 1E).

NDP-MSH treatment did not show any effect on TH+ cell count and striatal dopamine level in *Mc1r*^*e/e*^ mice treated with MPTP+LPS, suggesting that the protective effects of NDP-MSH are also mediated through MC1R (Figure 1F).

### BBB permeability in MPTP+LPS mice and penetration of NDP-MSH in brain

**Figure 2:**
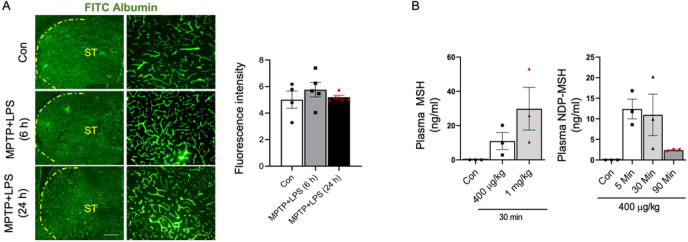
BBB permeability in MPTP+LPS mice and pharmacokinetics of NDP-MSH. C57BL/6 mice were treated i.p. with MPTP^.^HCl (20 mg/kg) and LPS (1 mg/kg) or vehicle (Con) from D1 to D4. Mice were sacrificed at 6 h and 24 h after the last dose, and FITC albumin assay was conducted. (A) Representative micrographs of FITC albumin and quantification of fluorescence intensity in striatum. Scale bars, 30 μm and 100 μm. C57BL/6 male mice (3–4 months old) were treated with MPTP^.^HCl (20 mg/kg)+LPS (1 mg/kg) and NDP-MSH from D1 to D4 and sacrificed at 5-, 30- and 90-min. Control (Con) mice received saline injection and sacrificed at 90 min. Levels of NDP-MSH in the plasma at (C) different doses and (D) different time points. Levels of NDP-MSH were below limit of detection (3.9 ng/ml) in the brain at all time points assessed. One-way ANOVA followed by Tukey’s post hoc test; n=3/group.

FITC albumin leakage assay showed that our MPTP+LPS regimen caused a slight breach in BBB permeability at 6 h but not at 24 h after the last dose (Figure 2A). We previously reported that NDP-MSH does not cross BBB in normal C57BL/6J mice (12). To evaluate brain penetration of NDP-MSH in MTPT+LPS model, we conducted LC/MS to assess NDP-MSH concentrations in the brain and plasma at different time points. While there were time- and dose-dependent increases in NDP-MSH concentrations in the plasma (Figure 2B), NDP-MSH in the brain was undetectable at all time points assessed. The absence of NDP-MSH in the brain could be explained by the insignificant disruption of BBB by the MPTP+LPS regimen, suggesting that the neuroprotective effects of NDP-MSH are mediated by its peripheral actions.

### NDP-MSH reduced inflammation in the periphery and the ventral brain

**Figure 3:**
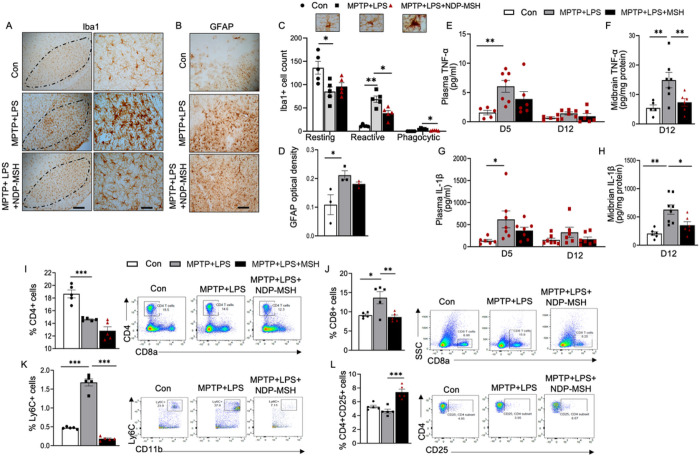
Systemic NDP-MSH treatment reduces neuroinflammation and modulates peripheral immune responses. C57BL/6 mice were treated i.p. with MPTP^.^HCl (20 mg/kg) and LPS (1 mg/kg) from day (D) 1 to D4 and NDP-MSH (400 μg/kg) or vehicle control from D1 to D12. Plasma was collected at D5 and D12. Mice were sacrificed at D12. (A) Cells stained positive for iba1 in the SN. Scale bars, 100 μm and 30 μm. (B) GFAP staining. Scale bar, 30 μm. (C) Morphological classification and quantification of iba1+ microglia. Two-way ANOVA followed by Tukey’s post hoc test; n=5/group. (D) Quantification of integrated optical density of GFAP in the SN. One-way ANOVA followed by Tukey’s post hoc test; n=3/group. ELISA assessment of TNF-α levels in (E) plasma at D5 and D12 and (F) ventral midbrain at D12 and IL-1β in (G) plasma at D5 and D12 and (H) ventral midbrain at D12. Two-way ANOVA followed by Tukey’s post hoc test. **p<0.01; n=5–7/group/time point. Flow cytometric analysis of the splenocytes showing percentages of (I) CD4^+^ helper T cell, (J) CD8^+^ cytotoxic T cells, (K) LY6C^+^ cell, and (L) CD4+CD25^+^ Tregs. One-way ANOVA followed by Tukey’s post hoc test; *p<0.05, **p<0.01; ***p<0.001; n=4–5/group.

Brain resident microglia staining positive for Iba1 were evaluated based on their morphological classification of iba1+ cells (Figure 3C). NDP-MSH reduced microglia activation and the number of reactive and phagocytic Iba1+ microglia in the nigral region (Figure 3A, 3C) but did not show effect on MPTP+LPS-induced gliosis (Figure 3B, 3D).

Peripheral inflammation is partly responsible for activated microglia in PD (43). We assessed inflammatory cytokines and found significantly higher concentrations of TNF-α and IL1-β in plasma and ventral midbrain of MPTP+LPS mice compared with the control group (Figure 3E, G). The increases in the levels of TNF-α and IL1-β in the brain were attenuated following treatment with NDP-MSH (Figure 3F, H). We also observed a decrease in levels of TNF-α and IL1-β at day 5 and day 12 in plasma. Although statistically non-significant, they pointed towards attenuation of peripheral inflammation markers by NDP-MSH.

To assess the status of different immune cells in the periphery, splenocytes were stained for markers of myeloid and lymphoid cells. We assessed MC1R expression in a pilot study and observed that MC1R was expressed on a number of immune cells from the spleen in normal C57BL/6J mice under basal conditions (Supplemental Figure 1). Exposure to MPTP+LPS caused a decrease in the percentage of CD4+ helper T cells and an increase in the percentage of CD8+ cytotoxic T cells (Figure 3I, J), consistent with previously implicated increased percentage of circulating cytotoxic T cells in PD (56). NDP-MSH treatment in MPTP+LPS mice significantly increased cytotoxic CD8+ T cells percentage (Figure 3J). Ly6C^high^ monocytes, which have also been reported to be involved in neuroinflammation (57) were significantly increased in MPTP +LPS mice, and the change was reversed in mice treated with NDP-MSH. Additionally, despite a decrease in the percentage of total CD4+ cells, a significant increase in the percentage of Tregs was observed in NDP-MSH treated mice (Figure 3L) compared to the untreated MPTP +LPS group. These results indicated possible involvement of Tregs, at least in part, in NDP-MSH impact on inflammation and neuroprotection.

### Depletion of Treg cells limited the neuroprotective effect of NDP-MSH

**Figure 4:**
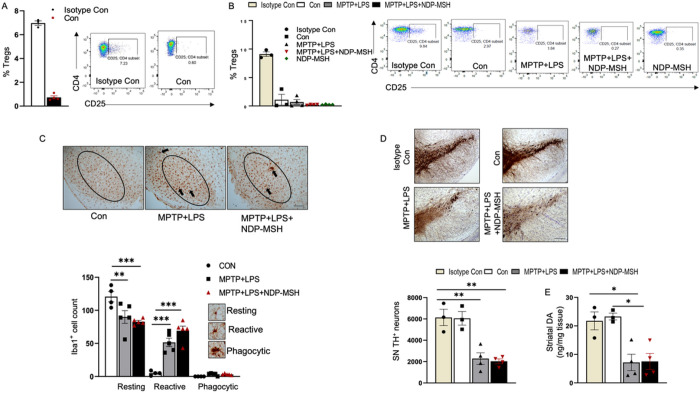
Depletion of Tregs abolishes neuroprotective effect of NDP-MSH. (A) C57BL/6 mice were pre-injected with PC61/CD25 antibody or isotype control. Mice were then injected with MPTP^.^HCl (20 mg/kg)+LPS (1 mg/kg) or vehicle (Con) and treated with NDP-MSH (400 μg/kg) or vehicle. Mice were sacrificed, and flow cytometry was carried out to assess the percentage of Treg cells in the spleen (A) before random grouping and (B) at the end of the treatment paradigm. (C) Representative micrograph staining for iba1 and morphological classification and quantification of iba1+ microglia in SN. scale bar 100 μm; n=4/group. Two-way ANOVA followed by Tukey’s post hoc test; **p<0.01; ***p<0.001. (E) Representative micrograph of TH+ staining and stereological quantification of TH+ cells in SN. Scale bar, 100 μm; n=34/group. One-way ANOVA followed by Tukey’s post hoc test. **p<0.01. (E) Striatal dopamine content; n=3–4/group. One-way ANOVA followed by Tukey’s post hoc test. *p<0.05. Representative results from 2–3 independent experiments, which were conducted using preserved samples or fresh samples and were not pooled.

Mykicki et al. have shown induction of Treg cells following treatment with NDP-MSH in EAE (17). In the present study we observed a significant increase in the percentage of Tregs following administration of NDP-MSH in MPTP+LPS mice (Figure 3L). To evaluate whether Tregs may be involved in NDP-MSH neuroprotection, we used PC61/CD25 antibody to deplete Tregs in mice. Mice were pretreated with the antibody, and then randomly grouped and treated with MPTP+LPS and NDP-MSH as described above. Depletion of Treg cells was confirmed before the randomization and at the end of the experiment among the treatment groups (Figure 4A, B). NDP-MSH failed to attenuate activation of Iba1+ microglia in MPTP+LPS mice lacking Tregs (Figure 4C). We did not observe any protective effects on TH+ cell count and striatal dopamine levels in Treg depleted mice treated with MPTP+LPS and NDP-MSH (Figure 4D, E), suggesting that neuroprotective effects of NDP-MSH are dependent on peripheral Tregs.

## Discussion

MC1R is found in both the peripheral and CNS, implying that both the peripheral and central forms of MC1R might potentially impact dopaminergic neurons in Parkinson’s disease. We previously reported MC1R-dependent neuroprotection of locally injected NDP-MSH in brain against αSyn dopaminergic neurotoxicity(12). The present study demonstrates that peripherally administered NDP-MSH protects dopaminergic neurons in a combined MPTP and LPS mouse model of PD. Intraperitoneal NDP-MSH improved behavioral performance in the pole test, and attenuated loss of nigral TH+ cells and striatal dopamine induced by MPTP+LPS. The dopaminergic neuroprotective effects are associated with significantly tempered microglia activation and reduced pro-inflammatory cytokines.

Our findings add to the growing evidence of the beneficial neuroprotective influence of peripherally administered NDP-MSH (15,17,58) in models of various neurological disorders. The levels of circulating α-MSH reportedly decline in patients with subarachnoid hemorrhage (58). Fu et al. reported ameliorative effects of NDP-MSH on oxidative stress and apoptosis in the affected neurons in mouse models of intracerebral hemorrhage (59). Mykicki et al. reported that NDP-MSH injected intravenously ameliorates neuroinflammation and EAE progression via signaling through orphan nuclear 4a receptor (Nr4a). Although NDP-MSH can bind to other MCRs, it has highest affinity to MC1R (60). Using *Mc1r*^*e/e*^ mice, Mykicki et al. further demonstrated that the beneficial effect of NDP-MSH is mediated by MC1R (17). Wu et al. reported similar MC1R mediated attenuation of neuroinflammation via CREB/Nr4a1/NF-κB pathway following i.p. injection of NDP-MSH (18). Our study similarly demonstrated the requirement of MC1R for NDP-MSH dopaminergic neuron protection. Together with previous studies from our group and others, our findings support the role of MC1R in neuroinflammation and dopaminergic neurodegeneration in PD. However, the involvement of other MCRs cannot be excluded. In a transgenic mouse model of AD, the most common age-related neurodegenerative disease, NDP-MSH intraperitoneal induced neurogenesis and cognitive recovery, and an MC4R antagonist abolished the beneficial effects of NDP-MSH (15). Further studies are needed to elucidate possible involvement of other MCRs in mediating NDP-MSH mediated dopaminergic neuroprotection in models of PD.

NDP-MSH is a relatively large peptide with no indications of brain penetrability in either normal mice (12) or in the MPTP+LPS model of PD. In the abovementioned studies using animal models of hemorrhage and EAE, which are known to have disrupted BBB, peripherally injected NDP-MSH restored BBB integrity (17,18,58). A disruption in BBB permeability has been reported in postmortem studies in PD patients (61) and in models of PD (44,62). The most common causative factors for this disruption have been proposed to be oxidative stress and neuroinflammation. A study with an MPTP mouse model of PD reported transient leakage of serum proteins and immune cells from the brain vasculature due to increased BBB permeability (62). We observed extravasation of FITC-albumin at 6 hours after last dosing of MPTP+LPS but not at 24 hours’ time point. Furthermore, NDP-MSH was not detectable at any of the timepoints we assessed. These results suggest that NDP-MSH likely exerted neuroprotective effects through its peripheral actions in our present model system.

The peripheral targets of NDP-MSH include specific immune cell populations. NDP-MSH reversed the MPTP+LPS-induced increases in percentages of monocytes and cytotoxic CD8+ T cells. Studies have reported increased infiltration of cytotoxic CD8+ T cells and little or no change in CD4+ T cells in PD patients and in MPTP models (56,63) of PD. Monocytes have also been implicated in PD pathogenesis. PD blood monocyte populations have more proliferative capacity compared to healthy controls (64). Ly6C^high^, the proinflammatory subset of monocytes, are increased peripherally in αSyn transgenic mice (65), and LPS animal models also exhibit increased infiltration of Ly6C^high^ monocytes which consequently leads to increased levels of TNF-α and IL-1β (66–68). We found increased levels of TNF-α and IL-1β in MPTP+LPS mice, and also the ability of NDP-MSH to ameliorate the changes in cytokine levels in the ventral midbrain.

Tregs play a critical role in regulating immune tolerance and homeostasis. Treg dysregulation has been implicated in PD, and adoptive transfer of Tregs is neuroprotective in MPTP models of PD (69). MC1R signaling reportedly triggers the expansion of Treg by acting on dendritic cells (70). Auriemma et al. showed that MC1R activated tolerogenic dendritic cells that stimulated and expanded functional CD4+CD25+Foxp3+ Tregs (71). NDP-MSH has also been shown to induce functional Tregs in EAE models (17). In this study we found elevated levels of Tregs in response to NDP-MSH in MPTP+LPS treated mice. Tregs-mediated neuroprotection is reportedly a result of increased neurotrophins, reduced proinflammatory molecules, cytokines, and oxidative stress, and induced apoptosis in the M1 state of microglia (72,73). Upon depletion of these cells, NDP-MSH neither abrogated microglia activation nor showed protective dopaminergic neuroprotection in the MPTP+LPS model of PD. Future studies are needed to address how Tregs may directly or indirectly mediate the dopaminergic neuroprotective effects of peripherally administered NDP-MSH.

Our study has limitations. We focused on Tregs. CD8+, B cells, and monocytes were not analyzed in detail. CD8+ cells particularly appear to show the strongest MC1R signal. MCR signaling has been reported to transform CD4+ T effector cells into CD4+CD25+ Tregs (74) and reactive CD8+ cells in tolerogenic type in murine contact dermatitis (75,76). More studies should be conducted to explore CD8+ and other immune cell populations in the MPTP+LPS model and their responses to NDP-MSH. We assessed infiltration of the immune cells into the brain. However, due to significant cell death, the results were not clear. Although our data indicates the roles of cytokines, specifically TNF-α and IL-1β in mediating peripheral and CNS inflammation, how the peripheral immune responses to MDP-MSH improve CNS inflammation and dopaminergic integrity will need further investigation to better explain the link between peripheral and central effects of MDP-MSH.

## Conclusion

The present study demonstrates that NDP-MSH protects nigrostriatal dopaminergic neurons in the MPTP+LPS model of PD. The neuroprotective effects of NDP-MSH are likely mediated by its peripheral actions and are MC1R dependent. In addition, NDP-MSH protects against MPTP+LPS-induced immune dysregulation and inflammation. Tregs may be necessary in the protective effect of NDP-MSH.

Together with previous studies from our group and others, our study supports the role of peripheral or systemic MC1R and the peripheral immune system, particularly Tregs, in the pathophysiology of PD. It also supports peripheral MC1R activation as a therapeutic strategy for PD. NDP-MSH is an approved drug currently used to prevent skin damage from sun exposure in people with erythropoietic protoporphyria (13). Our demonstration that the peripheral actions of NDP-MSH can be sufficient to protect dopaminergic neurons supports a rationale for repurposing NDP-MSH as a disease-modifying agent for PD.

## Data Availability

The datasets used and/or analyzed during the current study are available at open-access repository through zenodo.com; DOI: 10.5281/zenodo.7383131

## Abbreviations

MCRs: Melanocortin receptors
MC1R: Melanocortin 1 receptor
α-MSH: Alpha-melanocyte stimulating hormone
SN: Substantia nigra
αSyn: Alpha-synuclein
MPTP: 1-methyl-4-phenyl-1,2,3,6-tetrahydropyridine
AD: Alzheimer’s disease
BBB: Blood brain barrier
EAE: Experimental autoimmune encephalomyelitis
LPS: lipopolysaccharide
TH: Tyrosine Hydroxylase
GFAP: Glial Fibrillary Acidic Protein
HPLC-ECD: High-performance liquid chromatography with electrochemical detection
Nr4a: Nuclear 4a receptor
